# Different Tissue-Derived Stem Cells: A Comparison of Neural Differentiation Capability

**DOI:** 10.1371/journal.pone.0140790

**Published:** 2015-10-30

**Authors:** Gabriele Bonaventura, Sandrine Chamayou, Annalisa Liprino, Antonino Guglielmino, Michele Fichera, Massimo Caruso, Maria Luisa Barcellona

**Affiliations:** 1 Department of Pharmaceutical Science, Biochemistry Section, University of Catania, Catania, Italy; 2 Unità di Medicina della Riproduzione, Fondazione Hera, Sant’Agata Li Battiati (CT), Italy; 3 Department of Obstetrics and Gynecology and Radiological Sciences (OGiRA), University of Catania, Catania, Italy; 4 Department of Clinic and Molecular Biomedicine, University of Catania, Catania, Italy; 5 Institute of Neurological Sciences, Italian National Research Council, Catania, Italy; University of Nebraska Medical Center, UNITED STATES

## Abstract

**Background:**

Stem cells are capable of self-renewal and differentiation into a wide range of cell types with multiple clinical and therapeutic applications. Stem cells are providing hope for many diseases that currently lack effective therapeutic methods, including strokes, Huntington's disease, Alzheimer's and Parkinson's disease. However, the paucity of suitable cell types for cell replacement therapy in patients suffering from neurological disorders has hampered the development of this promising therapeutic approach.

**Aim:**

The innovative aspect of this study has been to evaluate the neural differentiation capability of different tissue-derived stem cells coming from different tissue sources such as bone marrow, umbilical cord blood, human endometrium and amniotic fluid, cultured under the same supplemented media neuro-transcription factor conditions, testing the expression of neural markers such as GFAP, Nestin and Neurofilaments using the immunofluorescence staining assay and some typical clusters of differentiation such as CD34, CD90, CD105 and CD133 by using the cytofluorimetric test assay.

**Results:**

Amniotic fluid derived stem cells showed a more primitive phenotype compared to the differentiating potential demonstrated by the other stem cell sources, representing a realistic possibility in the field of regenerative cell therapy suitable for neurodegenerative diseases.

## Introduction

Stem cells are present in every living organism. They are distinguished from the other cells because they are "unspecialized". Stem cells can reproduce indefinitely, giving rise at the same time both to stem cells and somatic cells designed to differentiate into cells of specific tissues and organs [1–7].

The interest in stem cells has increased enormously in recent years because they can differentiate into several lineages, including adipose cells, chondrocytes, osteoblasts, endothelial cells, and they are also suitable as neuronal cell sources for repair or regeneration of damaged central nervous system (CNS) structures [8–21]. However, cellular therapy based on CNS-derived neural stem cells has encountered many restrictions and difficulty of use in a clinical situation, due to their limited expansion ability in culture. In fact, while embryonic stem cells are totipotent, and have retained the ability to differentiate into all animal tissues, it is believed that adult stem cells have the limited ability to differentiate only into the cells of the tissue in which they reside [22–27].

An increasing number of scientific discoveries seems to challenge this classical dogma, suggesting that the ability of stem cells to generate a daughter cell is not limited to mature cell types present in the tissue in which they reside but, surprisingly, they can have a wider range [23–28].

The first evidence for the plasticity of adult stem cells has emerged from the study on the hematopoietic system, using *in vivo* functional tests that use the properties of clonogenic hematopoietic immature cells: it was observed that transplanted bone marrow cells are able to give rise to "atypical" progeny and regenerate, even if at a rather low frequency, other tissues [28–32]. On the other hand, the adult bone marrow of several animal species (mouse, rat, human) is already known to contain immature cells such as mesenchymal stem cells (MSCs) capable of generating multiple cell lines [2, 33–36].

Regarding Bone Marrow *mesenchymal stem cells (BM-MSCs)*, previous published reports on in vitro studies [4, 24–37] have shown a high potential for expansion, good genetic stability, compatibility with tissue engineering, as well as high *reparative* capacity of vital organs and tissues [38–40]; they are also able to develop into other cells, such as hepatocytes, cardiomyocytes and neural cells (both neurons and glial cells), [19, 41–50] although, it is not currently known how the differentiation of these cells *in vivo* happens [51–54].

Also mesenchymal stem cells from perinatal tissues (cord blood and amniotic fluid) are particularly useful for our purposes. These cells have been successfully differentiated into specialized cells from the three germ layers and therefore can be described as pluripotent stem cells [55–57]. Furthermore, these cells having been conserved for later stages of life, have found application for autologous transplantation, for foetuses and newborns suffering from genetic disorders.

Specifically it has been shown that cord blood mesenchymal stem cells (CB-MSCs) can differentiate into several lineages [58–61] and can be an example of multipotent or even pluripotent stem cells.

Although they have similar cellular, morphological and differentiation properties to the bone marrow mesenchymal stem cells they show advantages over bone-marrow cells, since the latter decrease in number and differentiation potential with age [62–64].

Amniotic fluid has also been the object of our attention because it contains multiple cell types derived mainly from exfoliating surfaces of the developing foetus such as cells from the foetal skin, respiratory system, urinary and gastrointestinal tracts, along with populations of MSCs. [65–69]. The uniqueness of these types of cells is their freshness. The characterization of this multipotent stem cell population, designated as amniotic fluid-derived stem cells (AFS), was initially described by De Coppi et al. [70]. AFS cells are characterized by high capacity for self-renewal and by their ability to differentiate towards lineages, representative of all three germ layers. Given these characteristics we also explored this source for its capability of differentiating into neural like cells.

The existence of stem cells with previously unappreciated differentiation potential has been recently challenged by evidence of a novel source of mesenchymal stem cells: the human endometrium, a highly regenerative tissue undergoing monthly cycles of growth, differentiation and shedding during a woman’s reproductive years [71–73]. It has been stated that adult stem or progenitor cells are responsible for the cyclical regeneration of the endometrial functional layer each month. [71, 74–75] As human endometrial stem cells are lightly isolated, they expand rapidly, without leading to technical problems by producing a clonogenicity higher than bone marrow and cord blood mesenchymal stem cells. [76]

The extremely limited self-repairing capacity of adult neural tissue justifies the search for new sources of cells and the need for strategies of intervention in neurodegenerative diseases as well as in the treatment of post-traumatic and hereditary diseases.

The aim of our work was to induce, by comparing, the differentiation process capability of adult and perinatal stem cells in neural cells from different sources such as bone marrow, umbilical cord blood, human endometrium and amniotic fluid, by analyzing similarities and differences and by hypothesizing future therapeutic uses. We tested the expression of neural markers such as GFAP, Nestin and Neurofilaments using the immunofluorescence staining assay and typical cluster of differentiation as CD34, CD90, CD105 and CD133 by using cytofluorimetric assay.

## Materials and Methods

Our mesenchymal stem cells sources were: bone marrow (BM), umbilical cord blood (CB), human endometrium (hE) and amniotic fluid (AF). In order to reduce individual variability among the recruited population, homogenous in sex, age and, where necessary, in the sampling site, stem cell samples from three donors were pooled. The same protocol was followed for all the stem cell sources analyzed. Written informed consent was obtained from all the patients. This study has been reviewed and approved by an Institutional Review Board (IRB) of Azienda Ospedaliero-Universitaria "Policlinico-Vittorio Emanuele" (Catania). The donors/patients are anonymous; their details are known only to the doctor who took the sample and takes care of their written consent following the ethical statement. The Patients were also informed and agreed for the use of their samples for research purposes

### 2.1 Bone marrow stem cells

Bone marrow samples (3ml) were obtained by aspirates directly from the red marrow in the iliac crest, under general anesthesia after the informed written consent was obtained. The fraction of mesenchymal bone marrow mononuclear cells was isolated with a density gradient centrifugation by using Ficoll-Paque PLUS at a ratio as follows: 1ml of the sample in phosphate-buffered saline solution (PBS) added by 2% Foetal Bovine Serum (FBS) in 1.5 ml of Ficoll (GE Healthcare Bio‐Sciences Corp., Piscataway, NJ, USA). The obtained mononuclear cells fraction, present to interphase at a density between 1,053 and 1.073 g/ml [77–79], was cultured in polystyrene coated flasks (Invitrogen Corp., Carlsbad, CA, USA) at a concentration of 2x10^5^/cm^2^, in the following basic medium: Dulbecco’s Modified Eagle’s Medium (DMEM) supplemented with 20% FBS and 1% antibiotic–antimycotic (Gibco, USA), incubated at 37°C in a humidified atmosphere containing CO2 at 5%. The non-adherent cells (as macrophages and lymphocytes) were discarded after 72 h of culture, and the adherent cells (as multipotent mesenchymal stem cells) were incubated in a fresh medium for an additional 4 days. When the flasks were 90% confluent, the cells were trypsinized by 0.05% trypsin solution, added by 0.53 mM EDTA at 37°C for 5 min, washed and resuspended in the basic medium, as previously defined.

The neural differentiation was achieved by adding to the cells, suspended in the basic medium and maintained for 6 days in *in vitro* culture, (DIV), the following mixed solution: 1mM dibutyryl cAMP (dbcAMP), 0.5 mM Isobutyl Methyl Xanthine (IBMX), 20 ng/ml human Epidermal Growth Factor (hEGF), 40 ng/ml basic Fibroblastic Growth Factor (bFGF), 10 ng/ml Nerve Growth Factor (NGF) and 10 ng/ml Brain-Derived Neurotrophic Factor (BDNF) (all reagents were purchased from Invitrogen Milan Italy), and monitored, for neural phenotype, by light microscopy at 2, 6 and 10 DIV.

### 2.2 Umbilical Cord Blood stem cells

Human cord blood MSCs were isolated from umbilical cord blood, donated from local maternity Hospitals with the donor’s written consent, by a standardized procedure using 10 ml syringes containing heparin as anticoagulant. Briefly, human umbilical cord blood was collected from three normal, full-term infants, delivered by caesarean section.

The mesenchymal cord blood mononuclear cells were isolated by density-gradient centrifugation (1.077–1080 g/ml) using Ficoll-Paque PLUS (GE Healthcare Bio‐Sciences Corp., Piscataway, NJ, USA). The interphase containing mononuclear cells was washed three times with PBS, centrifuged at 250 g for 10 minutes and then resuspended in PBS.

The collected cells were resuspended in DMEM/F-12 containing 20% FBS, 10ng/ml Epidermal Growth Factor (EGF; Sigma), and 1% antibiotic–antimycotic. The cells were plated at a density of 1.0×10^6^cells/cm^2^ in non-coated T-25 flasks (Beckon-Dickinson Milano, Italy) and maintained in a humidified atmosphere at 37°C under 5% CO2 in air.

The neural differentiation was achieved by adding to the cells suspended in the basic medium and maintained 10 DIV in the following mixed solution: 1mM dbcAMP, 0.5 mM IBMX, 20 ng / ml hEGF, 40 ng / ml bFGF, 10 ng/ml NGF and 10 ng/ml BDNF and monitored, for neural phenotype, by light microscopy at 2, 6 and 10 DIV.

### 2.3 Human Endometrium stem cells

Human endometrial tissue, was collected either from ovulating women, aged 36–53 years, undergoing endometrial biopsy for non-endometrial benign pathologies at the Gynaecology Department of Vittorio Emanuele Hospital, University of Catania or from oocyte’s donors at the infertility clinic *Unità di Medicina della Riproduzione in Sant’Agata Li Battiati* (CT), Italy. The obtained tissue fragments were mechanically treated and digested with 0.5% (wt/vol) collagenase I (Warthington Biochemicals Corporation, Lakewood NJ, USA). Stromal single cell suspensions were layered over Ficoll-Paque PLUS at a density-gradient centrifugation (1.077–1080 g/ml) [80] and centrifuged to remove red blood cells. The medium/Ficoll interface, mainly containing stoma cells and peripheral blood mononuclear cells, was carefully aspirated. The obtained cell fraction was cultured in 25 cm^2^ flasks (Invitrogen, Milan Italy) at a concentration of 2x10^5^/cm, in the following basic media: DMEM supplemented with 20% FBS and 1% antibiotic–antimycotic, incubated at 37°C in a humidified atmosphere containing CO2 at 5%.

Up to 90% cell confluence, the neural differentiation was achieved by adding to the cells suspended in the basic medium and maintained for 10 DIV the following mixed solution: 1mM dbcAMP, 0.5 mM IBMX, 20 ng/ml hEGF, 40 ng / ml bFGF, 10 ng/ml NGF and 10 ng/ml BDNF and monitored, for neural phenotype, by light microscopy at 2, 6 and 10 DIV.

### 2.4 Amniotic Fluid stem cells

Human amniotic fluid (AF) was obtained by ultrasound-guided amniocentesis performed on pregnant women for routine prenatal diagnosis purposes at gestational ages ranging from the 18th to the 22nd weeks. Using a 22G needle and under ultrasonographic control, 10 ml of AF was aspirated under aseptic conditions. The procedure is considered part of the standard diagnostic work used to rule out major chromosomal and genetic defects in some foetal disorders.

The specimens were centrifuged at 700g for 15 minutes, and the pellets removed and resuspended in 2 ml of DMEM supplemented with 20% FBS.

The total pool of cells was then plated in a 25 cm^2^ flasks. Cells were fed daily with DMEM supplemented with 20% FBS, 5 ng/ml fibroblast growth factor (FGF) (Promega, Milan Italy), 1% glutamine solution, 1% antibiotic-antimycotic, in a 95% humidified air, with 5% CO2 at 37°C for 48 hours; after that they were inspected for cell attachment, the medium was replaced, and non-adherent cells were removed.

The neural differentiation was achieved by adding to the cells suspended in the basic medium and maintained in culture for 10 DIV the following mixed solution: 1mM dbcAMP, 0.5 mM IBMX, 20 ng/ml hEGF, 40 ng/ml bFGF, 10 ng/ml NGF and 10 ng/ml BDNF and monitored, for neural phenotype, by light microscopy at 2, 6 and 10 DIV.

### 2.5 Immunochemical assay

To assess the differentiation which occurred, neural markers such as Glial Fibrillary Acidic Protein (GFAP) used as a marker to distinguish astrocytes from other glial cells during development, Nestin a member of the intermediate filament protein family expressed primarily in nerve cells and Neurofilaments commonly used as a biomarker of neuronal axons and dendrites, were tested by immunocytochemical staining procedures. The scraped cells from bone marrow, cord blood, human endometrium and amniotic fluid were fixed on the cover slips and exposed to 4% paraformaldehyde in 100mM PBS for 30 min and incubated overnight in the primary antibodies: mouse anti-GFAP monoclonal antibody (1:500, MAB 360, Chemicon-Millipore, Vimodrone, Milan, Italy) mouse anti-Nestin monoclonal antibody (1:500; CUB 7402, NeoMarkers, Freemont, CA, USA) mouse anti-Neurofilaments monoclonal antibody. Then, cover slips were incubated in the secondary antibodies, goat anti-mouse antibody IgG, conjugated with Fluorescein Isothiocyanate (FITC) to visualize nestin and neurofilaments expression (FITC 1:100; AP124F, Chemicon-Millipore, Vimodrone Milan, Italy). GFAP anti-mouse antibody conjugated with Cyanine Isothiocyanate (Cy3) (1:200; Jackson ImmunoResearch, Laboratories Inc., Suffolk, UK) to visualize GFAP expression. After that, cover slips were washed, mounted in PBS/glycerol (50:50 vol/vol), placed on glass microscope slides and analyzed on a Leica DM-RE fluorescent microscopy (Solms, Germany). For negative controls, primary antibodies were omitted and the same staining procedure was followed.

### 2.6 Cytofluorimetric Assay

Experiments to determine CD15, CD24, CD29, CD34, CD44, CD90 CD105 and CD133 [80–85], expression on neural-like cells coming from the different tissue sources as bone marrow, cord blood, human endometrium and amniotic fluid, after differentiation procedures, were carried out on isolated differentiated cells. The cells were lightly trypsinized, washed and resuspended in PBS added by 0.1% bovine serum albumin, and incubated for cell surface markers with the following anti-mouse primary antibodies: CD15, CD24, CD29, CD34, CD44, CD90, CD105, CD133 (all from Beckman Coulter Italia, Milan, Italy). After three washing procedures, the secondary antibody, anti-mouse IgG fluorescein-isothiocyanate (FITC)-conjugated, was added and incubated for 60 min at room temperature for CD 15 and CD34; for CD90, CD24 and CD29, the conjugated fluorescent antibody was cyanin (PC5) and for CD44, CD105 and CD133 the conjugated fluorescent antibody was phycoerythrin (PE). Three batches of control samples made by resuspended PBS cells from our selected sources were incubated only with the secondary antibody.

Our samples were analyzed using a Coulter Epics XL-MCL flow cytometer (Coulter Corporation, Miami, FL, USA). All shown data are given in percentage of CD15+, CD24+, CD29+, CD34+, CD44+ CD90+, CD105+, CD133+ cells. Control staining with FITC-coupled isotype-matched antibody were performed in preliminary experiment and never stained >0.3% of CD(34/90/105/133)^+^ cells.

At least 5,000 (forward and side scatter) gated events were collected per specimen. Cells were excited at 488 nm and the fluorescence was monitored at 525 nm for FITC signal, at 575 nm for PE signal, and at 675 nm for PC5. The fluorescence signals were collected using logarithmic amplification.

### 2.7 mRNA Isolation

Total RNA was extracted, from human mesenchymal stem cells coming from our selected tissue sources after neural differentiation procedures, using 1 ml of Tryzol reagent (Invitrogen Corp., Carlsbad, CA, USA) and then treated with recombinant DNase I/RNase free (Takara 1290 Terra Bella Ave.Mountain View, CA, USA). 200 μl of chloroform were added to the tubes and centrifuged at 12,000 rpm for 25 min at 4°C. The aqueous phase was precipitated with 1 vol 70% ethanol. The RNA was pelleted by centrifugation at 10,000 rpm for 1 min with Rnase-free water, and the RNA preparation was then stored at −80°C. RNA content was measured at 260 nm using a NanoDrop ND1000 spectrophotometer (NanoDrop Technologies). Total RNA (1 μg) was analyzed by real-time PCR.

### 2.8 Real-time PCR quantification

The quantitative real-time polymerase chain reaction (qRT-PCR) was performed with theTaqMan gene expression assay on an ABI Prism 7900 sequence analyzer according to themanufacturer’s recommended protocol (Applied Biosystems, Foster City, CA, USA). Each reaction was run in triplicate. The comparative threshold cycle (CT) method was used tocalculate the amplification fold as specified by the manufacturer. A value of 10 ng of reverse-transcribed RNA samples was amplified by using the TaqMan Universal PCRMaster Mix and TaqMan gene expression assay (Hs00909233_m1 for GFAP, Hs04187831_g1 for Nestin, Hs00193572_m1 for Neurofilaments, Applied Biosystem).

## Results

We divided the results pertaining to the microscopic imaging investigations, both by light microscopy (panel 1) and immunochemical assay (panel 2), in four sections reported as follows:

**1**) bone marrow **2**) umbilical cord blood **3)** human endometrium and **4**) amniotic fluid.

### 3.1 Bone Marrow

#### 3.1.1 Light microscopy observation

**Fig 1:**
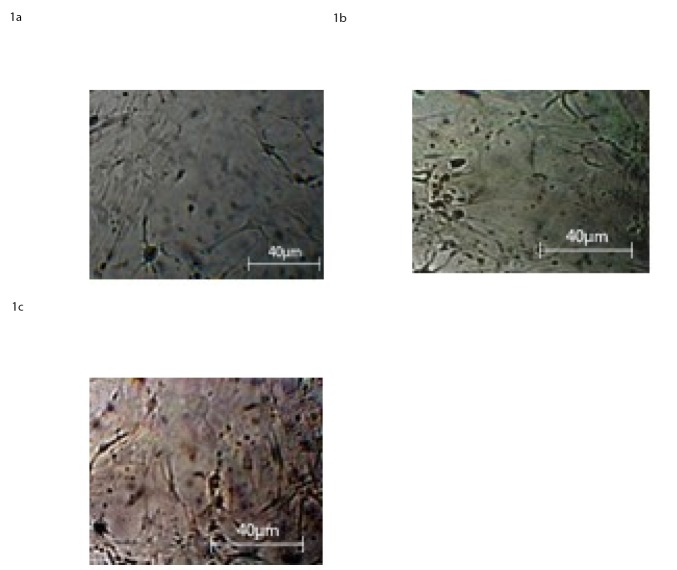
Light Microscopy images at 40X magnification of BM-MSC at 2 D.I.V a), 6 D.I.V. b), 10 D.I.V, c), in presence of neural differentiation factors as reported in the section: ‘Materials and Methods’.

BM-MSCs cultured in neural differentiation media were microscopically observed at 2, 6, and 10 DIV respectively. The cell culture at 2 DIV showed a neural-like appearance in only 30% of the cells “Fig 1A”. On the sixth day, 45% showed a typical neural morphology such as axon and dendrites “Fig 1B”. On the tenth day, about 60% of the cells developed dendrites and presented characteristic aspects of astrocytes and oligodendrocytes, together with few rounded neuron-like cells, “Fig 1C” “Panel 1”.

#### 3.1.2 Immunofluorescence staining assay

**Fig 2:**
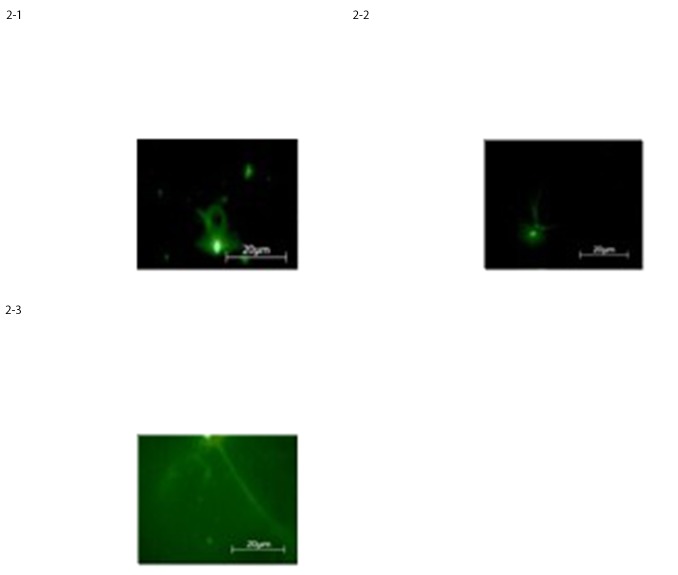
Expression of neural-like cells specific markers in differentiated BM-MSCs, at 10 D.I.V. evaluated by immunostaining for a) GFAP, b) Nestin, c) Neurofilaments, respectively.

To confirm the neural differentiation which occurred, as the microscopy images strongly suggested, we performed on cultured cells in differentiation media at 10 DIV an investigation on specific neural markers by using immunofluorescence staining assay. The immunostaining results showed that about 60% of the cells were positive for neural and glial markers such as GFAP, expressed by numerous cell types of the central nervous system, Nestin, a known marker of multipotent neural stem cells involved in the radial growth of the axon and Neurofilaments usually found in neurons, as a major component of the cytoskeleton cells (Fig 2).

### 3.2 Umbilical cord blood

#### 3.2.1 Light microscopy observation

**Fig 3:**
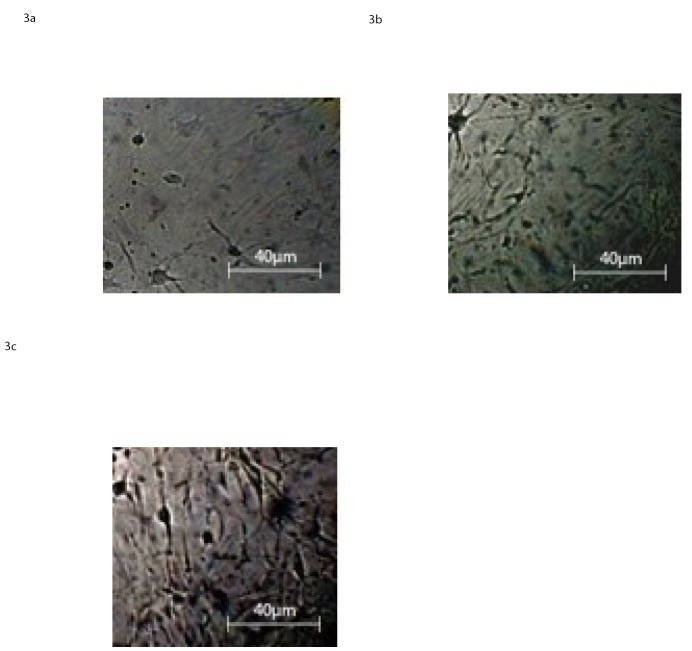
Light Microscopy images at 40X magnification of CB-MSC at 2 D.I.V a), 6 D.I.V. b), 10 D.I.V, c), in presence of neural differentiation factors as reported in the section: ‘Materials and Methods’.

The light microscopy images of the differentiated CB-MSCs in the temporary period of days in culture are shown in Fig 3A–3C.

Typical neural morphology progressively increasing after 2, 6 and 10 days in culture, neural factors supplemented, was presented by roughly 30%, 50% and 70% of the cells, respectively. More specifically, after two DIV, our cells exhibit the stage of neural-lineage precursor cells and subsequently, at the sixth day, two apparent different cell populations: glial cells, as astrocytes and oligodentrocytes and a few, rounded, small bipolar cells, neuron-like.

#### 3.2.2 Immunofluorescence staining assay

**Fig 4:**
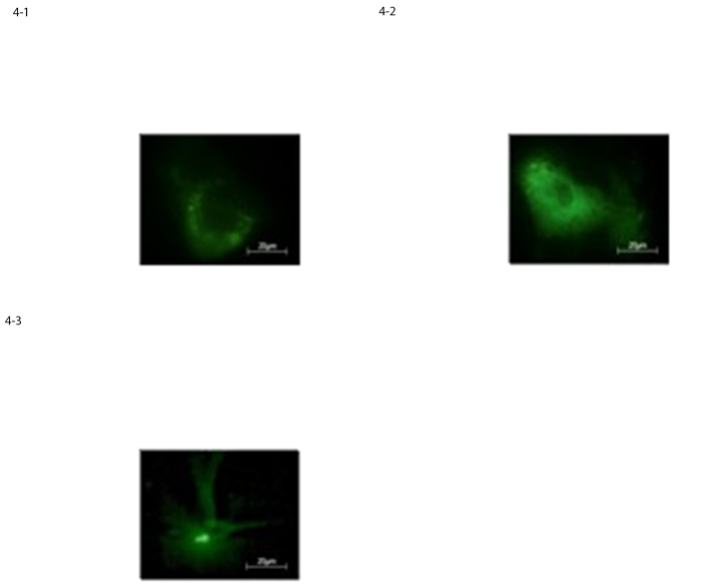
Expression of neural-like cells specific markers in differentiated CB-MSCs evaluated by immunostaining for a) GFAP, b) nestin c) Neurofilaments, respectively.

Immunofluorescence staining of cord blood-mesenchymal stem cells after 2, 6 and 10 DIV confirmed the microscopy observations; in-fact at 10 DIV roughly 70% of the cells was positive for early neural and glial cell markers as GFAP, Nestin and Neurofilaments, Fig 4A–4C.

### 3.3 Human endometrium

#### 3.3.1 Light microscopy observation

To enlarge the source availability of adult stem cells, we explored the possibility of obtaining mesenchymal stem cells from scraped human endometrial tissue.

We focused our attention on this peculiar source since it exhibits a tremendous regenerative ability and undergoes extraordinary growth in a cyclic manner by offering a double potential strategies: to study their role in cell-based therapies for regenerating tissues and to understand the involvement of the he-MSCs in abnormal endometrial proliferation. Our interest is concentrated on the first possible application.

**Fig 5:**
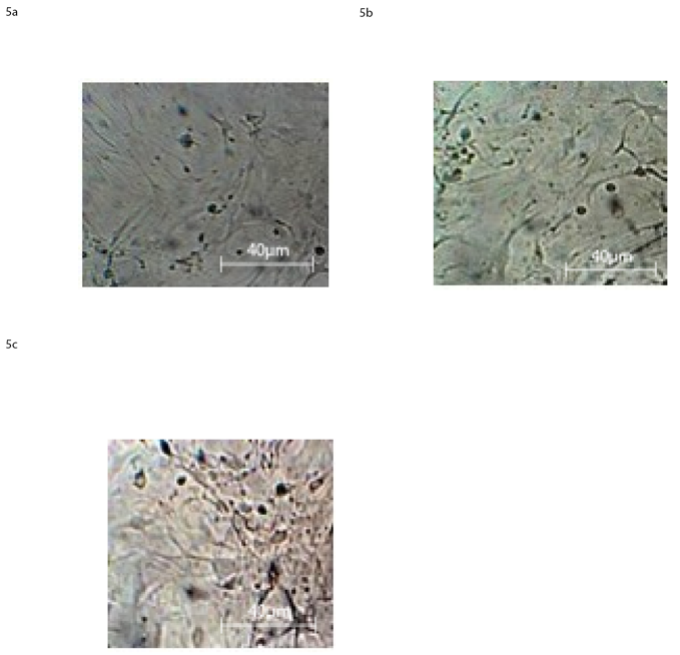
Light Microscopy images at 40X magnification of hE-MSC at 2 D.I.V a), 6 D.I.V. b), 10 D.I.V. c), in presence of neural differentiation factors as reported in the section: ‘Materials and Methods’.

hE-MSCs cultured in the neural differentiation media and microscopically observed at 2, 6 and 10 DIV showed a percentage of neural-like cells of about 30% at the second day a), 50% at the fourth day b) and 60–70% at the sixth day c) in vitro, “Fig 5A–5C”.

#### 3.3.2 Immunofluorescence staining assay

**Fig 6:**
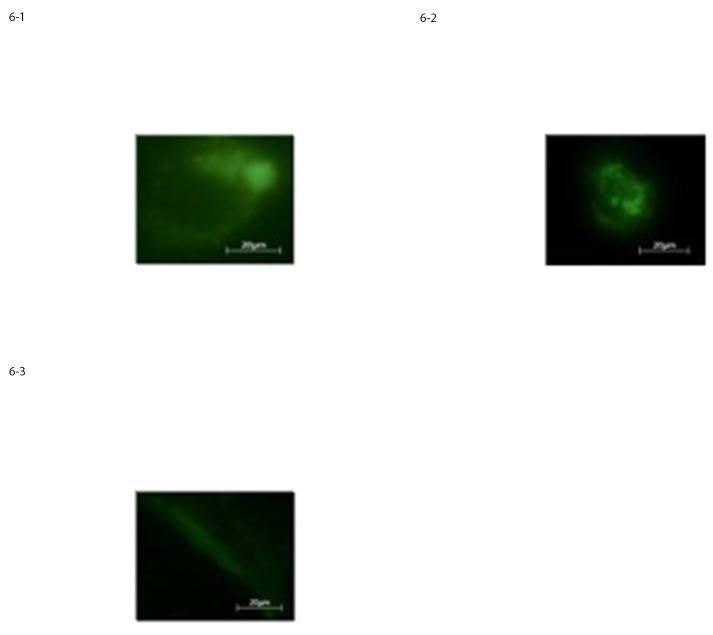
Expression of neural-like cells specific markers in differentiated he-MSCs assessed by immunostaining for a) GFAP, b) Nestin, c) Neurofilaments respectively.

The results obtained by the immunofluorence staining procedure for the he-MSCs at 10 DIV showed that the percentage of cells, positive for neural markers such as GFAP, Nestin and Neurofilaments was roughly the same as that revealed by the light microscopy observation, “Fig 6A–6C”.

### 3.4 Amniotic Fluid

#### 3.4.1 Light microscopy observation

**Fig 7:**
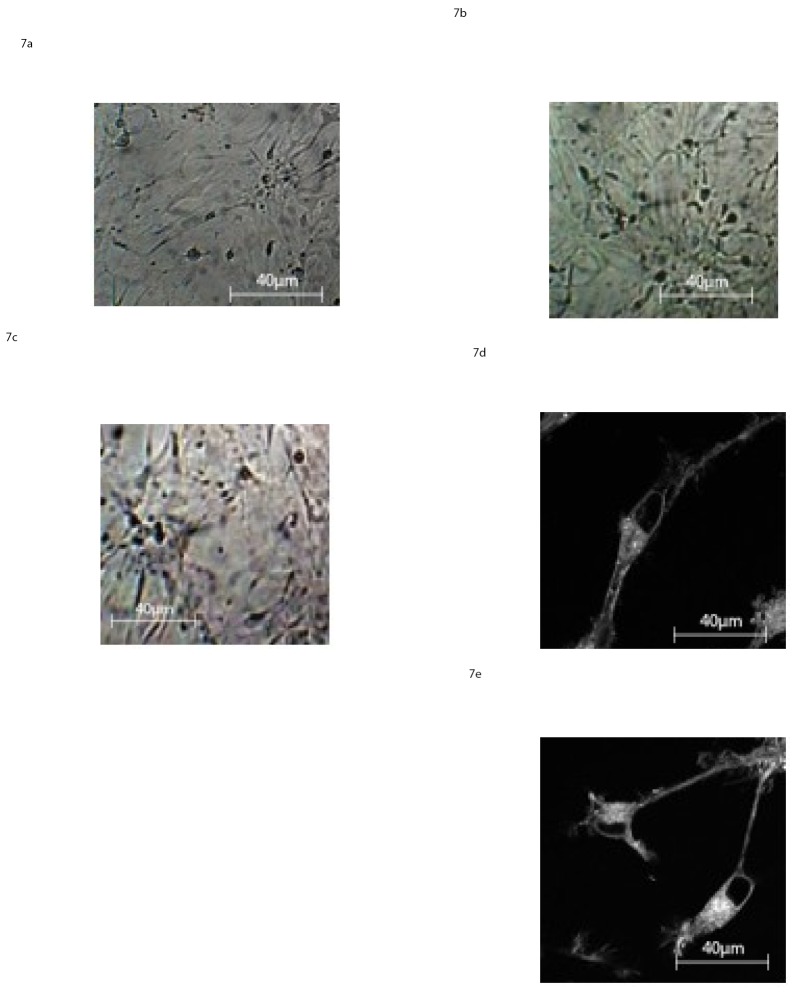
Light Microscopy images at 40X magnification of AF-MSC at 2 D.I.V a), 6 D.I.V. b), 10 D.I.V, c), in presence of neural differentiation factors as reported in the section: ‘Materials and Methods’d-e) Light Microscopy images at 100X magnification of AF-MSC at 10 D.I.V. in presence of neural differentiation factors as reported in the section: ‘Materials and Methods’.

AF-MSCs cultured under neural development conditions had changed their morphology already within the first 2 DIV as assessed by light microscopy examination. Two different cell populations appeared: the majority of the MSCs showed neural cell morphology represented by large flat cells and small bipolar cells. The bipolar cell cytoplasm retracts toward the nucleus, forming contracted multipolar structures, “Fig 7A–7D”. Over subsequent days, from the sixth day of in vitro culture, the cells display primary and secondary branches and cone-like terminal expansions, “Fig 7B”.

On the 10^th^ DIV, about 85% of the cells developed dendrites and presented typical characteristics of glia, (astrocytes), and neurons, “Fig 7C”.

These results show that AF-MSCs exhibit the best response to the neuro-differentiation procedures. To better characterize these observations we acquired more detailed images on a new split and plated cells pooled on glass bottom dishes one day prior the analysis. The images were performed by a Zeiss 710 microscope coupled to a Ti:Sapphire laser system (Spectra-Physics Mai Tai) equipped with a 40×1.2 NA, water immersion, lens (LUMPlanFl Olympus.), “Fig 7D and 7E”. These images clearly show bipolar shaped cells with apical and basal dendrites and cone like terminal expansions.

#### 3.4.2 Immunofluorescence staining assay

**Fig 8:**
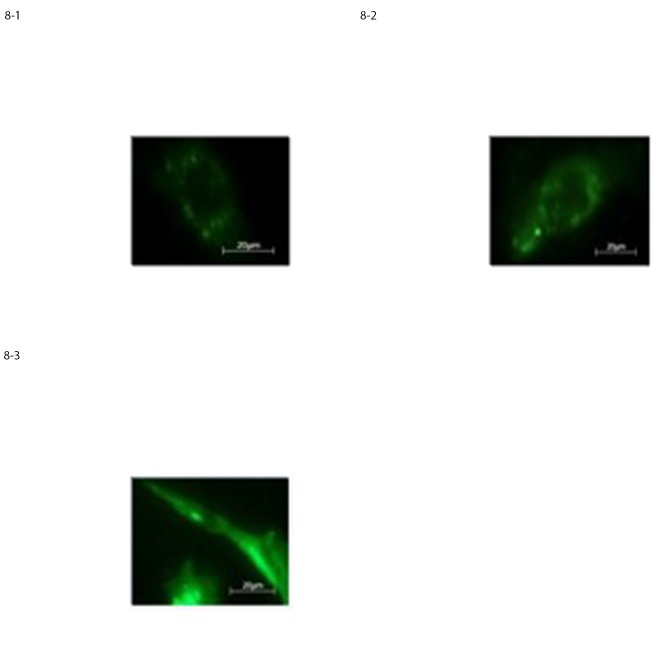
Expression of neural-like cells specific markers in differentiated AF-MSCs at 10 D.I.V. assessed by immunostaining for a) GFAP, b) Nestin, c) Neurofilaments, respectively.

Immunofluorescence staining of AF-mesenchymal stem cells after 2, 4 and 6 DIV, agreed with the cellular phenotype microscopically observed; in fact roughly 85% of the cells were positive for early neural and glial markers such as GFAP, Nestin and Neurofilaments. This high percentage demonstrated the different developmental stages of these cells compared with the other three stem cell sources under investigation “Fig 8A–8C”.

**Fig 9:**
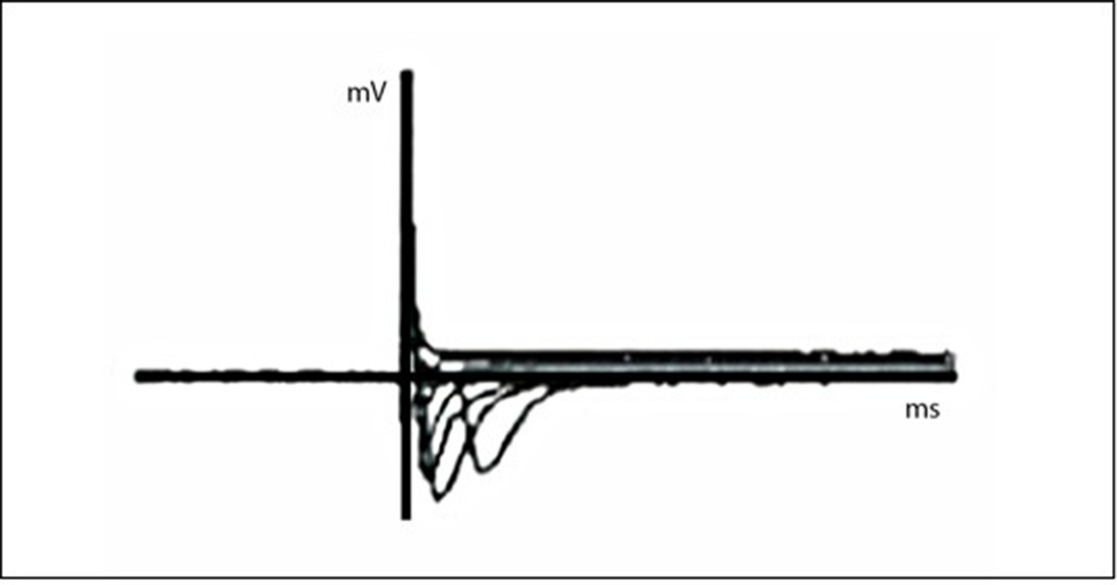
Na+ current traces evoked by a series 15mV voltage steps recorded from amniotic fluid differentiated neural-like stem cells.

To add a contribution to the already clear morphological characteristics, we tested a general aspect of the nervous cell functional properties. The differentiated neural-like stem cells from the amniotic fluid tissue source, which seemed the best source among those analyzed, were tested for their electrophysiological activity by the conventional whole-cell recording configuration. “Fig 9”.

We used whole-cell patch-clamp recordings to characterize electrophysiological properties of neural stem cells coming from amniotic fluid tissue source. The voltage-gated Na+ current is responsible for generating action potential in neural stem cells.

*In a canonical voltage-clamp experiment the cells were kept at -65mV and after that, a series of voltage steps up to +55mV, with an increment of 15 mV at a time, were applied. Na+ current traces, reaching the potentials seen in the previous cited procedure, show the excitatory cell characteristics seen in “Fig 9”.

#### 3.4.3 Light Microscopy quantitative analysis

**Fig 10:**
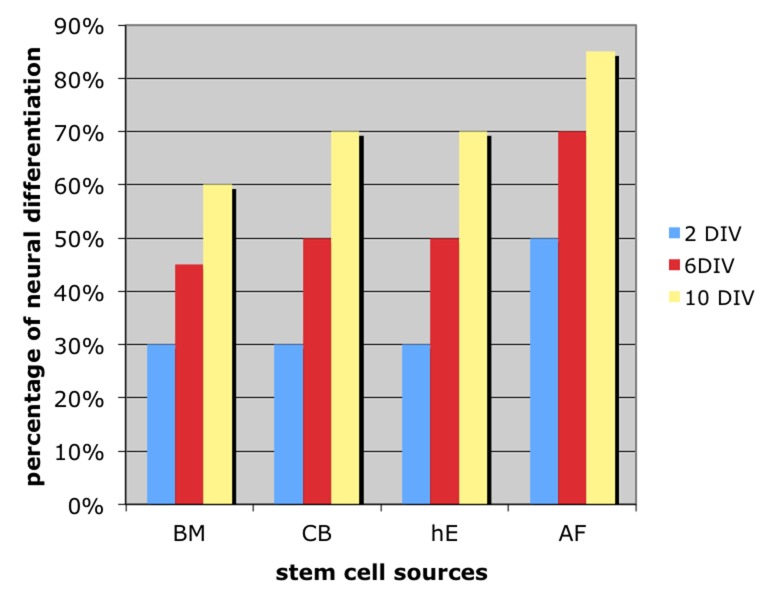
Quantitative analysis in form of histograms of the appearance of neural cell types morphology for each stem cell sources, at 2, 6, 10 days of culture in vitro.

In “Fig 10” is reported an overall quantitative analysis in form of histograms of the appearance of neural cell types morphology for each stem cell sources, at different days of culture in vitro.

**Fig 11:**
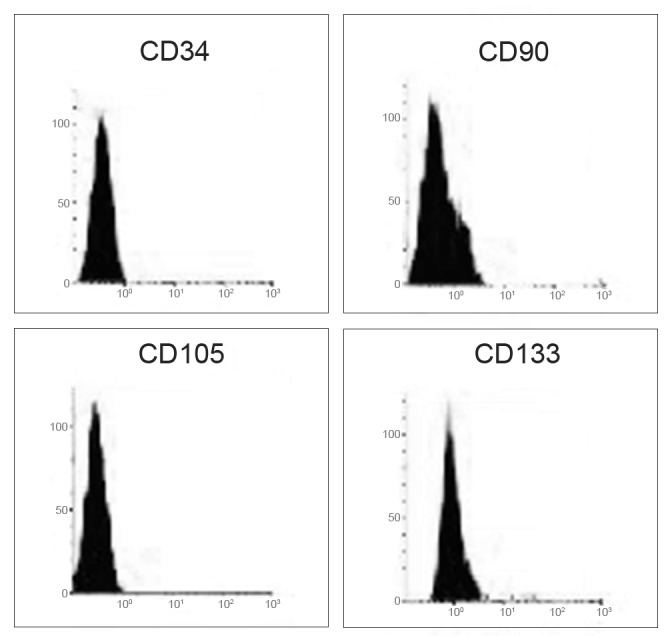
Immunostaining picture coupled with bright field image, obtained for the neural markers NESTIN, from AF-MSCs, 10 D.I.V. of neural differentiation treatment.

“Fig 11” shows light microscopy images of AF-MSCs after 10 D.I.V. of neural differentiation treatment coupled with immunostaining picture obtained for the neural markers NESTIN.

### 3.5 Cell identification by cytofluorimetric assay

**Fig 12:**
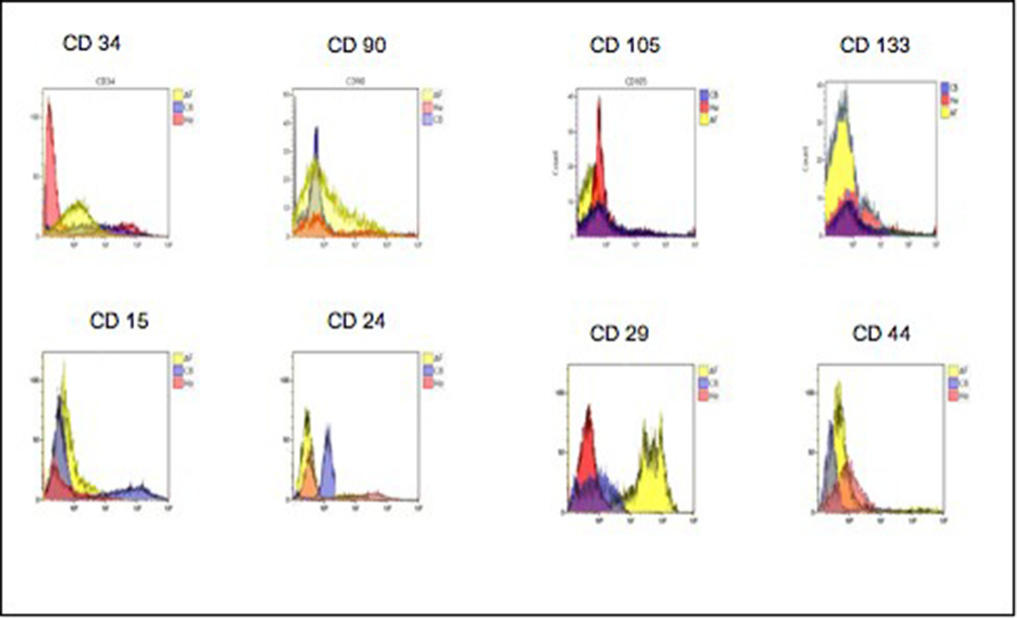
Representative flowcytometry diagrams of the CD34, CD90, CD105 and CD133 surface markers expression in mesenchymal stem cells from Bone Marrow. The fluorescence intensity as number of counts and the distribution diagram of positive cells are reported in ordinate and in abscissa respectively. Data represent means +/- SE of 3 independent experiments.

The cell phenotypic characteristics, surface topography, quantification, and internal structures of BM-MSCs, CB-MSCs, he-MSCs and AF-MSCs, after neural differentiation process, were detected by flow cytometry analysis, through the testing of CD34, CD90, CD105 and CD133 surface clusters expression. Some other clusters such as CD15, CD24, CD29, CD44 have been tested only to ascertain in our differentiated cells the chosen route. Flowcytometric diagrams are showed for these last CDs. All data are reported in “Fig 12”.

BM-MSC: Regarding the mesenchymal stem cells from bone marrow, after the addition of neural differentiation factors, their expression was negative for CD34, and positive at an extent of 18% and 28% for CD90 and CD133 respectively; this result was comforting for a neuronal cell type presence, even if glial cells too express CD90, but at a later stage of differentiation “Fig 12”.

**Fig 13:**
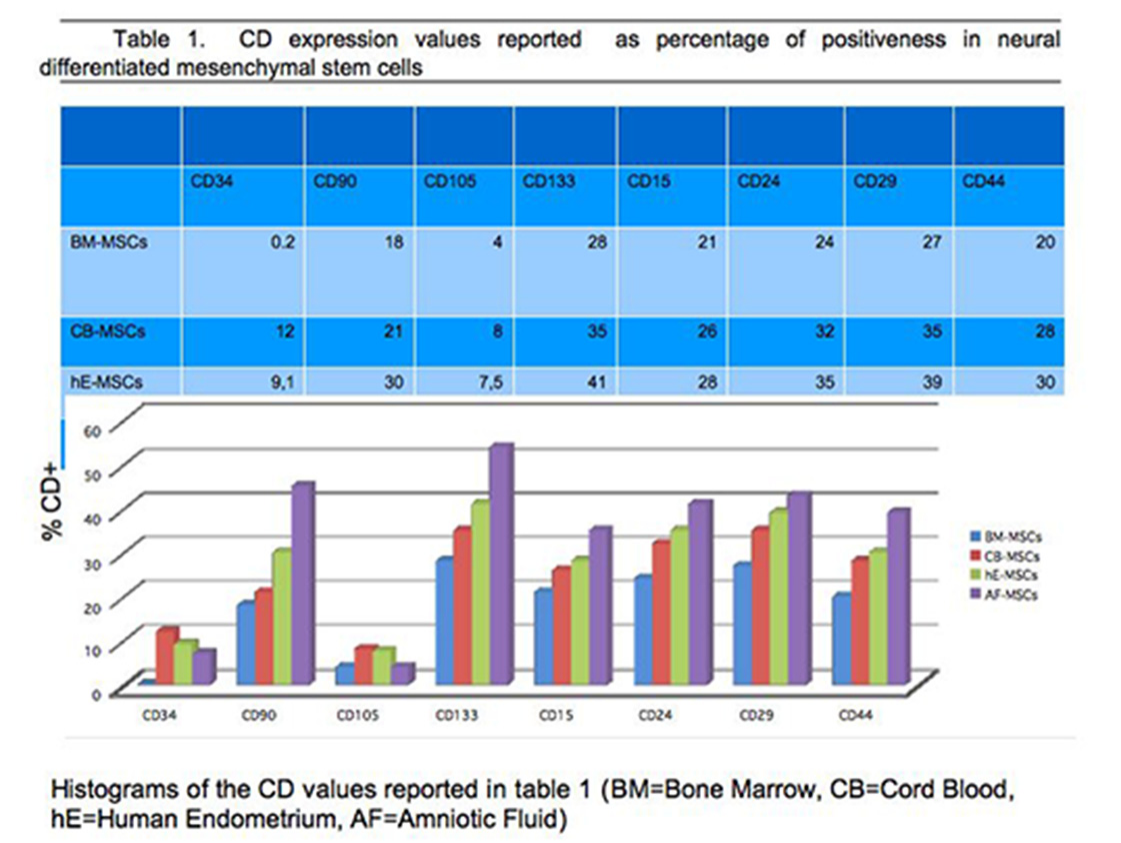
Representative flowcytometry diagrams of the CD34, CD90, CD105, CD133 CD surface markers expression in mesenchymal stem cells from, Cord Blood, Human Endometrium, Amniotic Fluid. The fluorescence intensity as number of counts and the distribution diagram of positive cells are reported in ordinate and in abscissa respectively. Data represent means +/- SE of 3 independent experiments.

**Fig 14:**
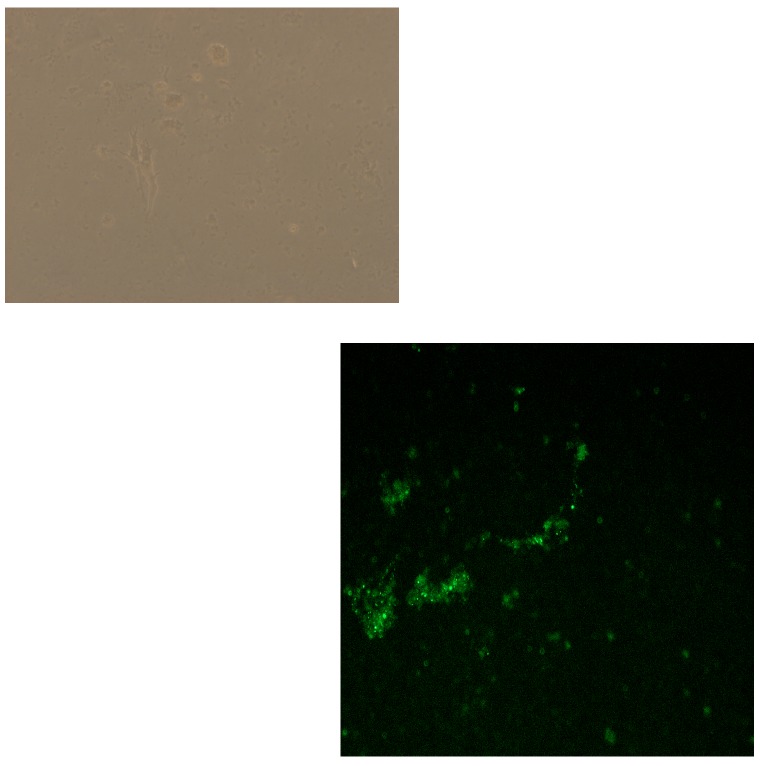
Value percentage of the expression of CD 34/90/105/133/15/24/29/44 for each stem cell source analyzed.

CB/hE/AF-MSCs: For the other MSC sources investigated, the obtained results on the same clusters of differentiation as those tested on BM-derived cells, are shown in Fig 10 panel A and panel B. Fig 12 shows the two dimensional density plots diagram for each stem cell source considered. By comparing the amniotic fluid-derived stem cells results with those from cord blood-derived, cultured in presence of the same neural differentiation factors, we observed that the expression of CD34 is lower for the stem cells amnio-derived (7%), compared to that of cord blood derived (12%); since we know that CD34, is expressed at high levels by the hematopoietic progenitor indicative of nonspecific immunity, the AF cells result suggests a hematopoietic maturation stage earlier than cord blood cells, and as a consequence a greater susceptibility (of the amnio derived stem cells), to the differentiation processes. This plausible possibility is also supported by the positivity of CD90 and CD133, the first being markers of axonal processes and the second being of neuronal and glial progenitor. We observed CD90^+^ in 45% of the amniotic fluid-derived stem cells compared to 21% in cord blood-derived stem cells and 54% compared to 41% for CD133^+^ respectively “Fig 12”, “Fig 13”, “Fig 14” “S1 Fig” “S1 Table”.

The human endometrium-derived stem cells (hE-MSCs) “Fig 12”, underwent the same neural differentiation process, exhibited for all the clusters examined the same pattern compared to the other stem cells but at a different percentage ratio and in particular 9.1%, 30% and 35% for CD34 CD90, CD133 compared to the results obtained for the same clusters from CB and AF. This result is indicative of an intermediate maturation stage, between the two types of mesenchymal stem cells from amniotic fluid and cord blood respectively.

We evaluated the expression of CD105, as a kind of negative control for all specimens examined.

CD105 is an endoglin, trans-membrane protein, expressed on vascular endothelial cells and marker of neovascularisation processes [86]. It is slightly expressed in amniotic fluid and in bone marrow (4%) and it is about double in cord blood and human endometrium derived stem cells (7.5%), contrary to the abundant presence in the umbilical cord tissue [87]

#### 3.5.1 RT-PCR

**Fig 15:**
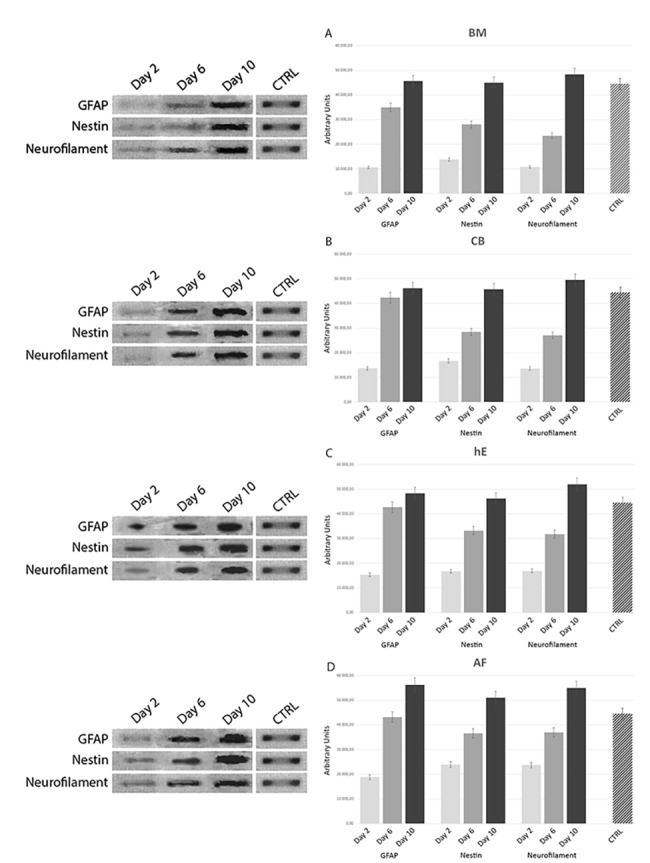
mRNA expression levels for classical neural genes such as GFAP, NESTIN and NEUROFILAMENTS, investigated with RT-PCR on BM-MSCs, CB-MSCs, he-MSCs and AF-MSCs, after 2, 6 and 10 D.I.V. after neural trans-differentiation treatment. Panel 1. Light Microscopy images at 40X magnification of BM-MSC (1a-c), CB-MSC (3a-c), hE-MSC (5a-c) AF-MSC (7a-c) at 2 D.I.V a), 6 D.I.V. b), 10 D.I.V, c), in presence of neural differentiation factors as reported in the section: ‘Materials and Methods’. Panel 2. Expression of neural-like cells specific markers in differentiated BM-MSC (2a-c), CB-MSC (4a-c), hE-MSC (6a-c) AF-MSC (8a-c) at 10 D.I.V. assessed by immunostaining for a) GFAP, b) Nestin, c) Neurofilaments, respectively.

The mRNA expression levels for classical neural genes such as GFAP, NESTIN and NEUROFILAMENTS, investigated with RT-PCR on BM-MSCs, CB-MSCs, he-MSCs and AF-MSCs, after 2, 6 and 10 D.I.V. after neural differentiation treatment, as reported in “Fig 15”, showed that Amniotic Fluid stem cell source present the highest levels of neural mRNA expression for our selected genes confirming what assessed by light microscopy observation, surface markers expression (CD), and immunostaining assay.

## Discussion

In this study we explored the neural differentiation competence of mesenchymal stem cells coming from different tissue sources. The ability of MSCs to differentiate into neural cells makes them potential candidates for the therapeutic replacement in neurological diseases. Stem cells are characterized *in vitro* by a high rate of growth and their differentiation, into different cell types, depends on numerous stimuli such as growth factors and extracellular matrix proteins.

Although the embryonic stem cells are considered the stem cells *par excellence*, because they are extracted in the earliest development stage of the embryo [88–91], they have a number of limitations. From the first announcement of human embryonic stem cell culture their use raised many controversial evaluations, especially in catholic countries. In our country ethical and legal restrictions have prohibited scientific research on human embryos. These obstacles have addressed the attention of several laboratories towards the research of alternative stem cell sources. It is widely accepted that bone marrow stroma cells are accessible from both healthy donors and patients and can be expanded on a therapeutic scale; for these reasons they have attracted attention for cell-based therapy. In the present study the BM-MSCs employment has been mainly investigated under two aspects: the capability, under specific stimulation, to differentiate into neural-like cell types and the comparison with other stem cell sources.

The obtained results confirm previous reports [92–94] providing evidence that BM-MSCs have the ability to differentiate into neural-like cells, when appropriately stimulated by specific growth factors present in the culture medium. Indeed, after 10 days of incubation in neural-differentiation media, approximately 60% of the cells presented typical nervous cell morphology confirmed by the positiveness for neural markers such as GFAP, nestin and neurofilaments. Our findings further support the evidence, previously reported in the literature, on the reliability of this source to differentiate into cell lines of different origin [95–101] Although two limitations, at least, have to be considered: firstly in the bone marrow mesenchymal stem cells are found only in low numbers [95] and secondly the proliferative potential and therefore the differentiation ability from older donors have a decreased lifespan associated with accelerated senescence, indicated by loss of proliferation under current culture conditions [102–103].

Umbilical cord blood -once thought capable only of turning into blood cells- can also be considered a viable alternative to human embryonic stem cells but more accessible compared to bone marrow and one of the most abundant sources of non-embryonic stem cells keeping in mind that the worldwide birth rate is over than 200 million per year [56–104]. In addition, unlike the collection of bone marrow, the umbilical cord blood collection is non-invasive and has no side effects on either the baby or the mother [104–106]. Moreover, stem cells from umbilical cord blood occupy an intermediate age stage between the embryonic stem cells and the adult stem cells (represented in our study by the bone marrow), which could lead to a higher proliferating potential than other somatic stem cells [107–108].

To demonstrate in a comparative manner the neural differentiation process of the CB-MSCs, we analyzed either cell morphology through the light microscopy and neural markers appearance by immunochemical staining test or the expression of cell-surface epitopes, such as CD90 and CD133 known neural stem cell markers by cytofluorimetric assay.

The results showed that the cell percentage, subjected to the differentiation process evaluated by the expression of CD90 and CD133 (21% and 31% for CB versus 12% and 28% for BM respectively), is only slightly higher for cord blood than that of the mesenchymal cells from bone marrow in the same cell culture conditions, that is reasonable evaluating the source’s derivation. But in spite of this result, the cord blood stem cells are a good candidate for a gradual replacement of mesenchymal stem cells because the bone marrow donation procedure is highly invasive and the differentiation potential decreases with increasing age.

A corollary is the limited number of hematopoietic progenitor cells in a single cord blood unit that can be considered a restriction for graft enhancement strategy but it is instead a good premise for the use of this MSC source in the differentiation processes.

By going in this direction we tested the differentiation ability of the human endometrial mesenchymal stem cells because the human endometrium, undergoing to an extraordinary growth in a cyclical manner, contains a population of stem cells, responsible for its regenerative ability. It has been demonstrated [109] that the endometrium regeneration is a consequence of cellular differentiation from stroma cells and not by direct extension from the basal epithelial layer. This confirms a shared origin with the bone marrow stroma cells but only a partial similarity. It has been also reported that oct-4 expression, tested by immunocytochemistry assay, in these cells is still high, so showing a preserved embryonic stage [109]. The endometrial stem cells properties include clonogenicity, proliferative potential and capacity for differentiation into one or more lineages [110]. In our case higher differentiation capacity of the human endometrial mesenchymal stem cells has been shown by the differences in the expression of neural phenotypic markers when compared to the result obtained by the BM-MSCs. This result finds support in previous research, concerning the involvement of neural basal medium supplemented factors such as bFGF and NGF in inducing endometrial stem cells to neural fate and specifically to cholinergic neurons [110–111] and in stimulating, via retinoic acid, neurite out-growth [112]. For these characteristics endometrial stem cells culture could be also employed as a model for the investigation of the neural cell development and regeneration molecular mechanisms.

Furthermore the observed differences in the expression of CD90 and CD133 (28% and 35% versus 12% and 28% respectively) and of CD105, reinforce this possible application. Although these differentiation clusters are described as associated with cell migration, it is not clear whether they are functionally important for homing capacities [113–114].

Nevertheless, our results obtained by searching for the ideal stem cell source allow us to indicate the amniotic fluid as the most promising source of human multipotent cells because, firstly it is not yet affected by differentiation stimuli, contrary to adult stem cells already confined in their permanent location [115–117] and secondly because these cells are routinely obtained utilizing minimally invasive technique, (*amniocentesis*), for prenatal diagnosis of foetal abnormalities.

In fact, human amniotic fluid-derived stem cells (hAFSCs) have attracted a great attention as an alternative cell source for transplantation and tissue engineering when compared with other stem and progenitor cell types [117]. These cells, derived from foetal tissues, have the ability to differentiate across all three germ layers [118–119], by maintaining the non-tumour forming properties of adult stem cells that is a typical problem associated with human embryonic stem cells.

Moreover, in contrast to hESCs, hAFSCs cells are not subjected to legal or ethical restrictions, nor are limited by lineage commitment characteristic of adult stem cells.

Our results have shown that amniotic fluid-derived stem cells have the greatest differentiation potential towards the neural cell lineage compared to the other tissue sources we examined, in terms of morphology and phenotype expression strongly positive for mesenchymal markers such as CD90 and negative for hematopoietic markers such as CD34.

Upon exposure to neural differentiation media AF-MSCs demonstrated morphological changes by adopting a large spherical-aspect associated with immunoreactive staining positiveness for the selected markers. The morphological changes were showed at a time of culture shorter than that of the other cells, derived from the compared tissue. This finding seems to show a more primitive phenotype against the differentiating potential demonstrated by the other cell sources.

The result that these cells are positive for CD133, 55%, marker of neuronal stemness, versus 41%, 35% and 28% for human endometrium, cord blood and bone marrow respectively, [120–121], is a strong indication for neurogenic differentiation. This result is also demonstrated by the positiveness of these cells to others neural patterns such as CD15, CD24, CD29 and CD44. In addition, hAFSCs proliferate more rapidly than post-natal somatic cells and embryonic stem cells, also maintaining prolonged undifferentiated proliferation, telomere length and a normal karyotype [118].

Furthermore, this neural precursor developmental capability is also evidenced by the mRNA expression analysis, performed with RT-PCR. Amniotic fluid mRNA investigated for GFAP, Nestin and Neurofilaments showed the highest expression levels, respect to the other stem cell sources analyzed under the same neuro-differentiation procedures for our neural-specific selected genes.

We would like to stress another advantage of using amniotic fluid when compared with other cell sources, that is: its enhanced plasticity as well as the feasibility of having autologous cell-based therapy available and ready to use either before or at the time of birth.

In conclusion our results show that amniotic fluid stem cells among the four stem cell sources taken into consideration represent an existing realistic possibility in the field of regenerative medicine [122–123] which makes them more suitable for research and therapeutic applications in neurodegenerative diseases, even though more work needs to be done to characterize in a more detailed manner the molecular mechanism which drives the differentiation.

## Supporting Information

S1 Fig Immunostaining biomarkers analysis vs quantitative expression of surface cluster. Supporting figure shows a comparison between the cluster of differentiation surface markers and the selected biomarkers for each stem cell source.
(TIFF)

S1 Table Phenotypic characteristics of the selected CDs. Supporting table shows the phenotypic characteristics identified by the expression of surface clusters, such as CD15, CD24, CD29, CD34, CD44, CD90, CD105 and CD133.
(DOC)

## Abbreviations

AF: amniotic fluid
AFS: amniotic fluid-derived stem cells
BDNF: Brain-Derived Neurotrophic Factor
bFGF: basic Fibroblastic Growth Factor
BM: bone marrow
CB: umbilical cord blood
CNS: central nervous system
dbcAMP: dibutyryl cAMP
DIV: days of *in vitro* culture
DMEM: Dulbecco’s Modified Eagle’s Medium
EGF: Epidermal Growth Factor
FBS: Foetal Bovine Serum
FGF: fibroblast growth factor
FITC: Fluorescein Isothiocyanate
GFAP: Glial Fibrillary Acidic Protein
hAFSCs: Human amniotic fluid-derived stem cells
hEGF: human Epidermal Growth Factor
hE: human endometrium
IBMX: Isobutyl Methyl Xanthine
MNCs: mesenchymal cord blood mononuclear cells
MSCs: mesenchymal stem cells
NGF: Nerve Growth Factor
PBS: phosphate-buffered saline solution
