# Supplementary figures and images for: Different Tissue-Derived Stem Cells: A Comparison of Neural Differentiation Capability
Source: PLoS One. 2015 Oct 30;10(10):e0140790. doi: 10.1371/journal.pone.0140790 (PMC4627815; doi:10.1371/journal.pone.0140790)

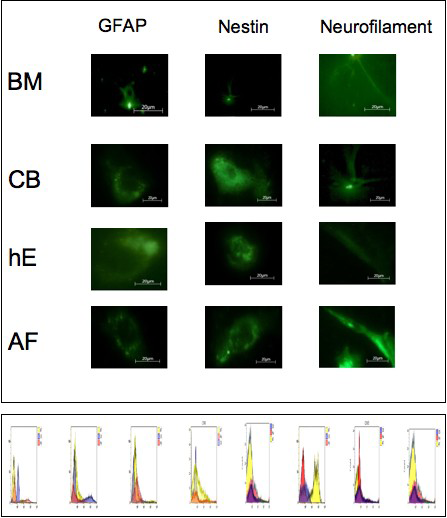

Supplement: S1 Fig — Supporting figure shows a comparison between the cluster of differentiation surface markers and the selected biomarkers for each stem cell source. (TIFF) [file pone.0140790.s001.tiff]
