# Supplementary material for: Different Tissue-Derived Stem Cells: A Comparison of Neural Differentiation Capability
Source: PLoS One. 2015 Oct 30;10(10):e0140790. doi: 10.1371/journal.pone.0140790 (PMC4627815; doi:10.1371/journal.pone.0140790)
Supplement: S1 Table — Supporting table shows the phenotypic characteristics identified by the expression of surface clusters, such as CD15, CD24, CD29, CD34, CD44, CD90, CD105 and CD133. (DOC) [file pone.0140790.s002.doc]

| CD15 | Surface cluster of differentiation expressed by neurosphere and neural precursor cells. |
| --- | --- |
| CD24 | Surface cluster of differentiation expressed by neural stem cells. |
| CD29 | Surface cluster of differentiation expressed by neural stem cells. |
| CD34 | Surface cluster of differentiation expressed by in the umbilical cord and bone marrow as hematopoietic cells, a subset of mesenchymal stem cells. |
| CD44 | Surface cluster of differentiation expressed by astrocyte precursor cells. |
| CD90 | Surface cluster of differentiation expressed by marker for a variety of stem cells and for the axonal processes of mature neurons. |
| CD105 | Surface cluster of differentiation expressed by monocytes, especially those transitioning into macrophages, low expression in normal smooth muscle cells, high expression vascular smooth muscle cells. |
| CD133 | Surface cluster of differentiation expressed by neuronal and glial stem cells. |
